# Anti-Inflammatory Effect of Charantadiol A, Isolated from Wild Bitter Melon Leaf, on Heat-Inactivated *Porphyromonas gingivalis*-Stimulated THP-1 Monocytes and a Periodontitis Mouse Model

**DOI:** 10.3390/molecules26185651

**Published:** 2021-09-17

**Authors:** Tzung-Hsun Tsai, Chi-I Chang, Ya-Ling Hung, Wen-Cheng Huang, Hsiang Chang, Yueh-Hsiung Kuo, Jong-Ho Chyuan, Lu-Te Chuang, Po-Jung Tsai

**Affiliations:** 1 Department of Dentistry, Keelung Chang-Gung Memorial Hospital, Keelung 204, Taiwan; tts1725@gmail.com; 2 Department of Biological Science and Technology, National Pingtung University of Science and Technology, Pingtung 912, Taiwan; changchii@mail.npust.edu.tw; 3 Department of Human Development and Family Studies, National Taiwan Normal University, Taipei 106, Taiwan; lucyh6022@hotmail.com (Y.-L.H.); wencheng7373@gmail.com (W.-C.H.); 4 Department of Biotechnology and Pharmaceutical Technology, Yuanpei University of Medical Technology, Hsinchu 300, Taiwan; hchang@mail.ypu.edu.tw; 5 Department of Chinese Pharmaceutical Sciences and Chinese Medicine Resources, China Medical University, Taichung 404, Taiwan; kuoyh@mail.cmu.edu.tw; 6 Chinese Medicine Research Center, China Medical University, Taichung 404, Taiwan; 7 Department of Biotechnology, Asia University, Taichung 413, Taiwan; 8 Hualien District Agricultural Research and Extension Station, Hualien 973, Taiwan; jonghoc@hdares.gov.tw; 9 Program of Nutrition Science, School of Life Science, National Taiwan Normal University, Taipei 116, Taiwan

**Keywords:** anti-inflammation, bitter melon, charantadiol A, *Porphyromonas gingivalis*

## Abstract

*Porphyromonas gingivalis* has been identified as one of the major periodontal pathogens. Activity-directed fractionation and purification processes were employed to identify bioactive compounds from bitter melon leaf. Ethanolic extract of bitter melon leaf was separated into five subfractions by open column chromatography. Subfraction-5-3 significantly inhibited *P. gingivalis*-induced interleukin (IL)-8 and IL-6 productions in human monocytic THP-1 cells and then was subjected to separation and purification by using different chromatographic methods. Consequently, 5β,19-epoxycucurbita-6,23(E),25(26)-triene-3β,19(R)-diol (charantadiol A) was identified and isolated from the subfraction-5-3. Charantadiol A effectively reduced *P. gingivalis*-induced IL-6 and IL-8 productions and triggered receptors expressed on myeloid cells (TREM)-1 mRNA level of THP-1 cells. In a separate study, charantadiol A significantly suppressed *P. gingivalis*-stimulated IL-6 and tumor necrosis factor-α mRNA levels in gingival tissues of mice, confirming the inhibitory effect against *P. gingivalis*-induced periodontal inflammation. Thus, charantadiol A is a potential anti-inflammatory agent for modulating *P. gingivalis*-induced inflammation.

## 1. Introduction

Periodontal diseases are complex, multifactorial diseases characterized by chronic inflammation of periodontal tissues, including gingival inflammation and alveolar bone resorption, and eventually tooth loss. Periodontitis begins as acute inflammation of the gingival tissue driven by polymicrobial infections and aggressive host immune and inflammatory responses via production of pro-inflammatory cytokines [1].

*Porphyromonas gingivalis*, a Gram-negative anaerobic bacterium, has been considered as a major oral pathogen in the development of chronic periodontitis [2]. *P. gingivalis* expresses several known virulence factors, such as lipopolysaccharide (LPS), fimbriae, proteases, and outer membrane vesicles [1]. Exposure to *P. gingivalis* causes innate responses through toll-like receptor (TLR)-2 and TLR-4 on the host cell surface and can trigger the production and release of pro-inflammatory mediators, such as interleukin (IL)-8 and IL-6, IL-1β, and tumor necrosis factor (TNF)-α. These pro-inflammatory cytokines play a significant role in the development of periodontitis [3]. IL-8 is produced primarily by gingival fibroblasts, gingival epithelial cells and endothelial cells. It is detectable in diseased periodontal tissues and has been associated with subclinical inflammation of the initial lesion [4]. Recently, IL-8 has been considered to be a potential therapeutic target for periodontitis [5]. IL-6 and IL-1β regulate inflammatory cell migration and osteoclastogenesis [4]. Since excessive secretion of pro-inflammatory mediators has been highly related to periodontitis pathogenesis, developing a strategy-based approach to suppress *P. gingivalis*-induced inflammatory responses may be a promising strategy for the alleviation of chronic periodontal disease.

Natural products from the herbal remedy, medicinal plants, functional foods, and their constituent have been considered to be effective in the prevention and treatment of periodontal diseases [6,7,8]. However, these studies did not purify specific compounds that have a meaningful anti-inflammatory effect on periodontitis from the crude extracts.

Bitter melon (*Momordica charantia*) exhibits several biological effects, such as antibacterial, antiviral, antidiabetic, hepatoprotective, immunomodulation, antioxidant, and anti-inflammatory activities [9,10]. Wild bitter melon (WBM; *Momordica charantia* L. var. *abbreviata* Seringe) is a wild variety of bitter melon. WBM fruit and young tender leaf are consumed as vegetables. Ethyl acetate extract of WBM fruits and its saponifiable (S) and nonsaponifiable (NS) fractions suppressed *Cutibacterium* *acnes* (formerly *Propionibacterium* *acnes*)-induced cytokine and matrix metalloproteinase (MMP)-9 levels in vitro and attributed the anti-inflammatory potential to phytol and lutein present in the NS fraction [11]. Polyphenol-enriched extract of WBM leaves effectively attenuates *C. acnes*-induced inflammatory responses by inhibiting infiltrations of neutrophils and IL-1β-expressing leukocytes in vivo. The anti-inflammatory activity of WBM leaf extract may be attributable to the phenolic and triterpenoid components [12]. Since WBM leaf extract exerts potent inhibitory activity against *C. acnes*-induced IL-8 production [12], its action against *P. gingivalis* was examined. We isolated 5β,19-epoxycucurbita-6,23-diene-3β,19,25-triol (kuguacin R) and 3β,7β,25-trihydroxycucurbita-5,23-dien-19-al (TCD) from WBM leaf extract and demonstrated that both compounds suppressed *P. gingivalis*-induced inflammatory responses with human THP-1 monocytic cell model and prevented periodontal disease progression in a mouse model of experimental periodontitis [13]. As part of our continuing efforts directed toward the discovery of bioactive compounds in WBM, 5β,19-epoxycucurbita-6,23(E),25-triene-3β,19(R)-diol (charantadiol A) was isolated from WBM leaf extract and its possible anti-inflammatory activity against *P. gingivalis* was evaluated in this study.

## 2. Results and Discussion

### 2.1. Effects of Sub-Fractions from Leaf Extract of Wild Bitter Melon and Charantadiol A on P. gingivalis-Induced Cytokines in THP-Cells

**Figure 1:**
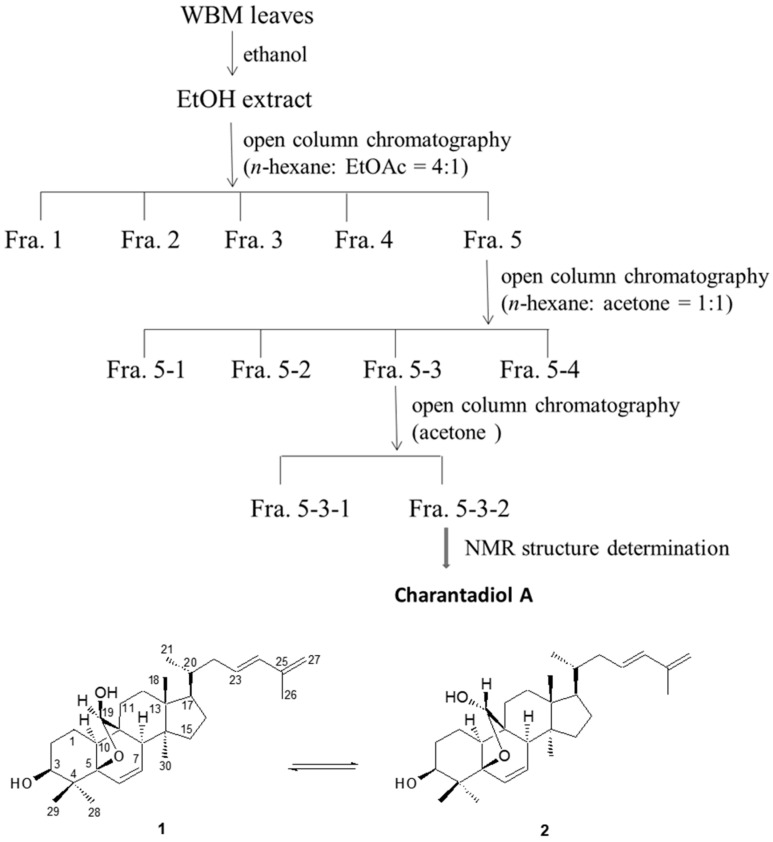
Schemes of the extraction and isolation of charantadiol A. Due to the C-19 hemiacetal carbon in charantadiol A (**1**), its 19(*S*) epimer (**2**) was also found as impurity in the NMR spectra.

Previous studies indicated that *P. gingivalis* can elicit high levels of IL-6 and IL-8 production in a variety of cell types comprising human oral epithelial cells, periodontal ligament cells and monocytes [13,14,15]. We previously demonstrated that sub-fractions, the fraction 5 (Fra. 5) and Fra. 5-2, isolated from crude WBM leaf extract inhibited *P. gingivalis*-stimulated IL-8 production by THP-1 cells [13]. In the present study, the Fra. 5-3 (Figure 1) was fractionated and evaluated to determine the fractions that contained effective substances. Then, co-culture model of heat-inactivated *P. gingivalis* and THP-1 monocytes was used to evaluate the suppress effects on *P. gingivalis*-induced inflammatory responses by the components of Fra. 5-3.

**Figure 2:**
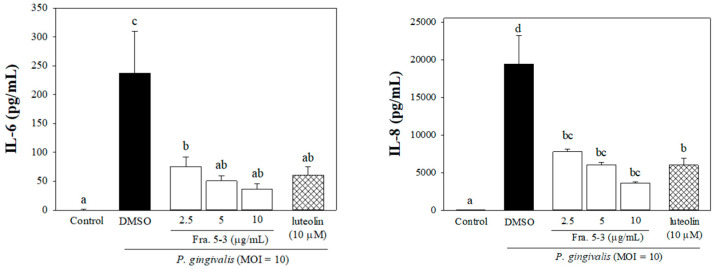
Effects of sub-fraction (Fra. 5-3) isolated from wild bitter melon leaf extract on *P. gingivalis*-induced pro-inflammatory cytokine productions in human monocytic THP-1 cells. Cells were incubated with 0.1% (*v*/*v*) DMSO (as a vehicle control), or co-cultured with *P. gingivalis* (M.O.I. = 10) and different concentrations of sub-fraction 5-3 (2.5, 5 or 10 μg/mL) for 24 h. The cell-free culture supernatants were subsequently collected and analyzed for the content of IL-8 and IL-6. Experiments were performed three times in triplicate. Each value represents the mean ± SD. Values with different letters are significantly different at *p* < 0.05.

To determine whether Fra. 5-3 would affect cell viability, THP-1 cells were incubated firstly in culture medium supplemented with various concentrations of tested samples. No adverse effect on cell proliferation was observed when the concentration of Fra. 5-3 was below 10 μg/mL by the 3-(4,5-dimethylthiazol-2-yl)-2,5-diphenyltetrazolium bromide (MTT) assay (data not shown). Figure 2 shows that the productions of IL-8 and IL-6 were significantly elevated in response to *P. gingivalis* stimulation. However, culture medium supplied with different concentrations of Fra. 5-3 significantly reduced respective cytokine production by as much as 85% (IL-6) and 81% (IL-8) (Figure 2).

**Figure 3:**
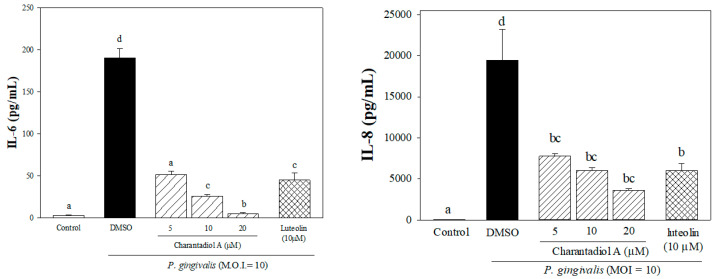
Effects of charantadiol A on *P. gingivalis*-induced pro-inflammatory cytokine production in human monocytic THP-1 cells. Cells were incubated with 0.1% (*v*/*v*) DMSO (as a vehicle control), or co-cultured with *P. gingivalis* (M.O.I. = 10) and different concentrations of charantadiol A (5, 10 or 20 μM) for 24 h. The cell-free culture supernatants were subsequently collected and analyzed for the content of IL-8 and IL-6. Experiments were performed three times in triplicate. Each value represents the mean ± SD. Values with different letters are significantly different at *p* < 0.05.

Moreover, charantadiol A, as mixtures of cucurbitane triterpenoid epimers, was isolated from Fra. 5-3 (Figure 1). No adverse effect on cell proliferation was observed when charantadiol A concentration was below 20 μM using the MTT assay (data not shown). In *P. gingivalis*-stimulated THP-1 cells, treatment of charantadiol A significantly suppressed *P. gingivalis*-induced productions of IL-6 (up to 97%) and IL-8 (up to 59%) (Figure 3).

Charantadiol A, a cucurbitane-type triterpenoid, is also a biological active component in bitter melon fruit and shows hypoglycemic effect in streptozotocin-induced diabetic rats [16]. Cucurbitane-type triterpenoids exert anti-inflammatory activity by inhibiting nitric oxide production in lipopolysaccharides (LPS)-stimulated RAW 264.7 macrophage cells [17]. In this study, we have demonstrated that charantadiol A is as potent as luteolin, a well-known antioxidant and anti-inflammatory flavonoid, in suppressing *P. gingivalis*-induced inflammatory responses in vitro (Figure 3).

### 2.2. Effect of Charantadiol A on TREM-1 mRNA Expression Level in P. gingivalis-Stimulated THP-1 Cells

**Figure 4:**
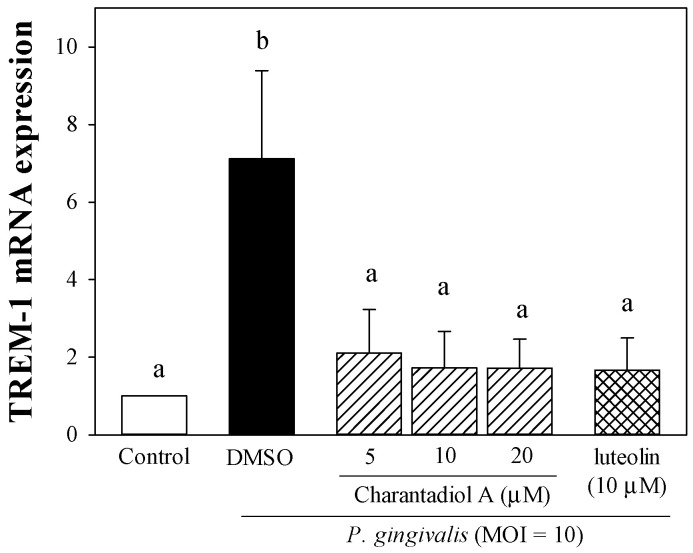
Effect of charantadiol A on *P. gingivalis*-induced triggering receptors expressed on myeloid cells-1 (TREM-1) mRNA expression. THP-1 cells were cultured with 0.1% (*v*/*v*) DMSO (vehicle control), or co-incubated with *P. gingivalis* (M.O.I. = 10) and different concentrations of charantadiol A (5, 10 or 20 μM) for 4 h to determine the TREM-1 mRNA levels. Experiments were performed three times in triplicate. Each value shows the mean ± SD. Values with different letters are significantly different at *p* < 0.05.

The triggering receptor expressed on myeloid cells-1 (TREM-1) is a cell surface receptor of the immunoglobulin superfamily expressed on polymorphonuclear leukocytes, monocytes, macrophages, dendritic cells, vascular smooth muscle cells, and is upregulated in the presence of inflammation to amplify pro-inflammatory cytokine production [18,19]. Co-triggering of TREM-1 and TLR4 results in a synergistic increase in TLR4-mediated pro-inflammatory cytokine and chemokine secretion [20]. Exposure of *P. gingivalis* induces significantly higher expression of TREM-1 mRNA and upregulates the expression of the TREM-1/DAP12 pathway in monocytes [21,22]. Willi and co-workers reported that periodontitis patients have higher TREM-1 gingival expression than healthy controls [23]. Doxycycline is used as an adjunct treatment in clinical periodontal therapy and has been shown to reduce *P. gingivalis*-induced IL-8 secretion by inhibiting TREM-1 expression and release [24]. Consistent with the previous findings [21,22,24], the TREM-1 mRNA level was significantly elevated in response to *P. gingivalis* (Figure 4). Treatments of charantadiol A significantly inhibited bacterially induced TREM-1 mRNA expression (Figure 4), and this effect may partly account for its anti-inflammatory property. Our present results show for the first time that charantadiol A downregulated *P. gingivalis*-induced TREM-1 expression. However, additional studies are needed to further support for the possible mechanisms underlying inhibitory effect of charantadiol A on pro-inflammatory cytokine production.

### 2.3. Effect of Charantadiol A on IL-6 and TNFα mRNA expression in P. gingivalis-Stimulated Gingival Tissue of Mice

**Figure 5:**
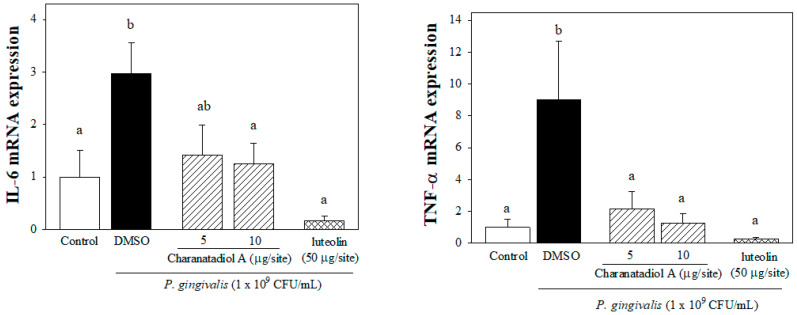
Charantadiol A suppressed pro-inflammatory cytokine mRNA expressions in *P. gingivalis*-stimulated gingival tissue of mice. Each value shows the mean ± SD. Values with different letters are significantly different at *p* < 0.05.

The pro-inflammatory cytokines, IL-1, IL-6, and TNF-α, appear to have central roles in periodontal tissue destruction [25]. IL-6 plays a crucial role mainly in the initiation and acute phase of periodontitis [26]. Additionally, IL-6 plays a role in the transition between acute and chronic inflammation, it enhances T-cell proliferation and accelerates of bone resorption by increasing osteoclast formation [27]. IL-6 is highly expressed in inflamed periodontal tissue and gingival crevicular fluid, which has been shown to be related to the severity of periodontitis [26,28]. TNF-α possesses a wide range of immune-regulatory functions to stimulate the production of chemokines or cyclooxygenase products, which consequently amplifies the degree of inflammation [27]. TNF-α has shown to participate in the initiation of periodontitis by injuring the oral mucosa barrier. Moreover, a high level of circulating TNF-α derived from periodontal tissue may contribute to systemic inflammation-associated diseases [26]. In this study, we showed that charantadiol A can affect immune responses in *P. gingivalis*-stimulated mouse gingival tissue. As shown in Figure 5, *P. gingivalis*–induced IL-6 and TNF-α mRNA expressions were attenuated by respective co-injection of charantadiol A (5 μg) or luteolin (50 μg).

The effectiveness of a conventional mechanical treatment against gingivitis is clear. However, topical adjunctive therapy with antimicrobials or anti-inflammatory agents has been applied for periodontal treatment [29]. Most natural products have been applied topically (as mouthwash, toothpaste, chewing gum etc.). Evidences show a beneficial effect of anti-inflammatory agents against gingivitis, either as a single treatment modality or as an adjunctive therapy [30]. Hence, it is worthy to investigate natural products which possess the beneficial effect on gingival inflammation. We previously described methods for isolating and purifying of kuguacin R and TCD and demonstrated their anti-inflammatory action in vitro and in vivo [13]. We showed that pro-inflammatory cytokine (IL-6 and IL-8) expression was induced by *P. gingivalis* infection but decreased by treatment with kuguacin R or TCD [13]. The activation of mitogen-activated protein kinase (MAPK), a signaling pathway for pro-inflammatory cytokines in periodontitis [31], was modulated by kuguacin R or TCD [13]. However, the yield of charantadiol A is lower than that of kuguacin R and TCD, making it difficult to acquire enough of an amount of charantadiol A to explore more details of its mechanism. Therefore, we are not able to make further analysis as we did on kuguacin R and TCD in our previous research. Nevertheless, the shortages of inexpensive, pure kuguacin R, TCD and charantadiol A are still limiting the exploration of their potentially beneficial applications to human health. Certainly, future investigations on the toxicological and pharmaceutical evaluation of these cucurbitane triterpenoids are expected.

## 3. Materials and Methods

### 3.1. Plant Materials

WBM (a cultivar of Hualien-1) leaves were obtained from the Hualien District Agricultural Research and Extension Station, Hualien, Taiwan. The fresh aerial parts of WBM were harvested. WBM leaves were collected and then a voucher specimen (number NTNUHung-2014-09) was deposited in the Department of Human Development and Family Studies, National Taiwan Normal University, Taipei, Taiwan. The voucher specimen of the plant was authenticated by Dr. Po-Jung Tsai, Professor, National Taiwan Normal University, Taipei, Taiwan. After cleaning with water, the WBM leaves were air-dried and ground using a blender. Powdered WBM leaves were stored in the dark at −20 °C until used.

### 3.2. Isolation and Determination of Charantadiol A

In this study, we further optimized our previous method for the preparation of WBM leaf extract [13]. Dried and powdered WBM leaves (100 g) were extracted twice with 2 L of ethanol (1:20, *w*/*v*) at room temperature on a rotary shaker at 200 rpm in the dark for 24 h. The blended mixture was then centrifuged at 5000× *g*. The supernatant obtained was filtered and then evaporated to dryness under reduced pressure (45–50 °C), yielding 14.6% of crude ethanol extract. As shown in Figure 1, crude ethanol extract was chromatographed on silica gel column chromatography eluted with n-hexane/ethyl acetate (EtOAc) (4:1) to give five sub-fractions (Fra.1~Fra.5). All samples of the Fra. 5 (25.7 g) were further chromatographed on a silica gel column (35 mm × 45 cm) eluted with n-hexane: acetone (Me_2_CO) (1:1) to further obtain four sub-fractions Fra. 5-1 (1.6 g), Fra. 5-2 (3.9 g), Fra. 5-3 (1.5 g), and Fra. 5-4 (17 g). Fra. 5-3 was further purified on silica gel with acetone to provides two sub-fractions, Fra. 5-3-1 and Fra. 5-3-2. Charantadiol A was obtained by purification of Fra. 5-3-2 using semi-preparative HPLC with a Lichrosorb Si gel 60 column (5 μm, 250 × 10 mm) eluted with CH_2_Cl_2_-EtOAc (7:1) at 2 mL/min (3.1 mg). Its chemical structure was identified by comparison of the spectroscopic data with those of published in the related references [32].

Nuclear magnetic resonance (^1^H NMR and ^13^C NMR) techniques were used for the structure elucidation of the compounds. NMR spectra were recorded on a Bruker spectrometer (400 MHz for ^1^H NMR and 100 MHz for ^13^C NMR) instrument and using CDCl_3_ as solvent (Supplementary Figure S1).

*5β,19-epoxycucurbita-6,23(E),25-trien-3β,19(R)-diol.* amorphous white powder; [α]25D-122.1° (c 0.2, CHCl_3_); IR (KBr) ν_max_ 3421, 3028, 2947, 2875, 1645, 1608, 1450, 1420, 1381, 1118, 1082, 983, 941, 879, 756 cm^−l^; ^l^H NMR (400 MHz, CDC1_3_) δ_H_: 6.10 (1H, d, J = 15.2 Hz, H-24), 6.07 (1H, dd, J = 2.0, 10.0 Hz, H-6), 5.64 (1H, dd, J = 4.0, 10.0 Hz, H-7), 5.61 (1H, m, H-23), 5.11 (1H, d, J = 8.0 Hz, H-19), 4.84 (2H, br s, H-26), 3.75 (1H, d, J = 9.6 Hz, 3-OH), 3.38 (1H, m, H-3), 2.81 (1H, br s, H-8), 2.67 (1H, J = 8.0 Hz, 19-OH), 2.46 (1H, t, J = 8.8 Hz, H-10), 1.82 (1H, s, Me-27), 1.19 (1H, s, Me-28), 0.89 (1H, d, J = 6.4 Hz, Me-21), 0.87, 0.86, 0.83 (each 3H, s, Me-29, Me-18, Me-30); ^13^C NMR (100 MHz, CDCl_3_) δ_C_: 14.7 (C-18), 17.3 (C-1), 18.7 (C-27), 18.7 (C-21), 19.7 (C-30), 20.5 (C-28), 23.1 (C-11), 24.0 (C-29), 27.1 (C-2), 28.0 (C-16), 30.5 (C-12), 33.5 (C-15), 36.6 (C-20), 37.2 (C-4), 39.8 (C-22), 40.6 (C-10), 41.4 (C-8), 45.1 (C-13), 48.0 (C-9), 48.5 (C-14), 50.2 (C-17), 76.0 (C-3), 86.6 (C-5), 105.4 (C-19), 114.1 (C-26), 129.2 (C-23), 132.4 (C-7), 132.7 (C-6), 134.2 (C-24), 142.2 (C-25); EI-MS *m*/*z* 408 [M-HCO_2_H]^+^ (9), 384 (11), 360 (5), 319 (4), 309 (6), 281 (16), 272 (17), 229 (13), 173 (11), 91 (29), 86 (72), 58 (100).

### 3.3. Cell Culture

The THP-1 cell line (BCRC 60430) was obtained from the Bioresource Collection and Research Center and maintained in RPMI 1640 (Gibco) supplemented with 10% heat-inactivated fetal bovine serum (FBS, Gibco), penicillin (100 U/mL), and streptomycin (100 μg/mL) at 37 °C in a humidified atmosphere with 5% CO_2_. A suspension of THP-1 cells (1 × 10^5^ cells/well) was cultured in 96-well culture plates with treatment of various concentrations of tested samples for 24 h at 37 °C in a humidified atmosphere of 5% CO_2_. The cell viability of THP-1 cells was determined by the 3-(4,5-dimethylthiazol-2-yl)-2,5-diphenyltetrazolium bromide (MTT; Sigma-Aldrich; St. Louis, MO, USA) assay described elsewhere [13].

### 3.4. Preparation of Heat-Inactivated P. gingivalis

The *P. gingivalis* strain BCRC14417 was obtained from the Bioresource Collection and Research Center, Hsinchu, Taiwan. Bacterial suspensions to induce periodontitis of mice were prepared by a method described elsewhere [13]. Briefly, *P. gingivalis* was cultured anaerobically in tryptic soy broth (TSB, Difco, Detroit, MI, USA) supplemented with 2.5% yeast extract, hemin, and menadione at 37 °C. The numbers of bacteria were determined with a spectrophotometer (at an optical density at 600 nm) based on a standard curve established by colony formation on bacterial plates. To prepare heat-inactivated *P. gingivalis*, bacterial suspensions in phosphate-buffered saline (PBS) were heated at 80 °C for 30 min, washed with PBS, and re-suspended in RPMI 1640 medium (Gibco, Carlsbad, CA, USA).

### 3.5. Stimulation of THP-1 Cells with P. gingivalis and Cytokine Measurements

Fra. 5-3 and charantadiol A were re-dissolved in dimethyl sulfoxide (DMSO; RDH Chemical Co., Spring Valley, CA, USA) to 20 mg/mL of stock solution for the sequential experiments. A well-established co-culture model of *P. gingivalis* and THP-1 cells was used to investigate the anti-inflammatory properties of WBM leaf extracts [13]. Briefly, THP-1 cells (2 × 10^5^ cells/well) were seeded in 96-well plates with serum-free medium and were stimulated with heat-inactivated *P. gingivalis* at multiplicity of infection (M.O.I.) of 10 (bacteria/THP-1 cell) alone or in combination with various concentrations of tested WBM extraction samples, DMSO (0.1%) as a vehicle control, and luteolin (Sigma, as a positive control) at 37 °C with 5% CO_2_ humidified atmosphere. After incubation for 24 h, the cell-free supernatants were collected, and the amount of IL-6 or IL-8 was determined using the respective enzyme immunoassay kits (Invitrogen, Carlsbad, CA, USA).

### 3.6. RNA Extraction of THP-1 Cells and Quantitative Real-Time Polymerase Chain Reaction (PCR)

THP-1 cells were cultured in 6-cm cell culture dishes (4 × 10^6^ cells/dish) for 24 h, and then co-incubated with heat-inactivated *P. gingivalis* (M.O.I. = 10) with various concentrations of tested samples (charantadiol A or luteolin). Cells were harvested and washed with PBS. Total RNA of human THP-1 cell samples was extracted and isolated with the TRIzol reagent (Invitrogen), according to the manufacturer’s instructions. cDNA was then synthesized from the RNA in a reaction mixture of oligo (dT) primers and reverse transcriptase (Promega, Madison, WI, USA), following the manufacturer’s instructions. Primers and probes were selected for the genes: GAPDH (glyceraldehyde-3-phosphate dehydrogenase) was used as the housekeeping gene. We used the forward 5′-CCA TAG GAG AGC AAC AGA-3′ and reverse 5′-GCC TCG TTC TAG TCA CAT ACA-3′ primers for triggering receptor expressed on myeloid cells (TREM-1), and the forward 5′-GTG AAG GTC GGA GTC AAC G-3′ and reverse 5′-TGA GGT CAA TGA AGG GGT C-3′ primers for GAPDH. These primer pairs amplified, respectively, a 106 bp fragment of the TREM-1 cDNA and a 112 bp fragment of the GAPDH cDNA. Real-time PCRs were conducted in an iCycler iQ Real-Time detection system (Bio-Rad, Hercules, CA, USA) using iQ^TM^ SYBR Green Supermix (Bio-Rad). The relative amounts of the PCR products were analyzed by iQ™5 optical system software (ver. 2.1; Bio-Rad). All expression levels were normalized using the GAPDH as an internal standard in each sample. Fold expression was defined as the fold increase relative to controls.

### 3.7. Effect of Charantadiol A on P. gingivalis-Induced Cytokine Expression In Vivo

We evaluated the protective effects of charantadiol A or luteolin on *P. gingivalis*-stimulated periodontal inflammation in a mouse model by using the method described elsewhere [13]. Six-week-old male C57BL/6 mice were obtained from the National Laboratory Animal Center (Taipei, Taiwan). The mice were housed in groups of 5 per cage, under standard temperature-controlled conditions with a 12 h/12 h light–dark cycle and free access to food and water throughout the experiments. All animal experiments were conducted in accordance with the Guide for the Care and Use of Laboratory Animals and were approved by the Animal Care Committee of the National Taiwan Normal University (IACUC Permit No. 103020). Throughout the period of the study, mice were fed with sterile standard solid mice chow diet and sterile water. Periodontitis was induced by an intra-gingival injection of heat-inactivated *P*. *gingivalis* according to the methods by Tsai et al. [13]. After 1 week of adaptation, animals were randomly divided into five groups (n = 5). Heat-inactivated *P*. *gingivalis* (1 × 10^9^ CFU in PBS) or PBS (as vehicle control) was injected once daily into the mandibular (lower inset) gingival tissues of mice for 3 days. To study the effects of charantadiol A or luteolin, they were respectively administered once daily for 3 days with co-injection of heat-inactivated *P*. *gingivalis* suspensions. After 14 days of bacterial injection, mice were then sacrificed with carbon dioxide asphyxiation. The gingival tissues were excised for the extraction of total RNA. *P*. *gingivalis*-induced IL-6 and TNF-α expression were determined by reverse transcription qualitative polymerase chain reaction (RT-qPCR) as previously described [13].

### 3.8. Statistical Analysis

All data are presented as means ± SD. Statistical analyses were performed using the SPSS 20.0 statistical package (Chicago, IL, USA). The data were evaluated for statistical significance with the one-way ANOVA followed by Duncan’s multiple range tests. A *p* value of <0.05 was considered statistically significant.

## 4. Conclusions

In conclusion, we have demonstrated that charantadiol A suppressed *P. gingivalis*--stimulated TREM-1 expression, thereby reducing the levels of pro-inflammatory mediators in THP-1 cells. Furthermore, charantadiol A exerted anti-inflammatory effect in periodontitis mimicking conditions in mice. Altogether, charantadiol A is an attractive cucurbitane for periodontitis treatments, and more investigations can be expected for further support the efficacy of charantadiol A on periodontitis.

## Data Availability

Data are contained within the article.

## Supplementary Materials

The following are available online. Supplementary Figure S1. Spectra of charantadiol A. ^1^H NMR (400 MHz) of charantadiol A (a), ^13^C NMR, DEPT-135, DEPT 90 spectra (100 MHz) of charantadiol A (b), EI-MS spectrum of charantadiol A (c), and IR spectrum of charantadiol A (d).

## Sample Availability

Samples of the compounds are not available from the authors.
