# Supplementary material for: Anti-Inflammatory Effect of Charantadiol A, Isolated from Wild Bitter Melon Leaf, on Heat-Inactivated Porphyromonas gingivalis-Stimulated THP-1 Monocytes and a Periodontitis Mouse Model
Source: Molecules. 2021 Sep 17;26(18):5651. doi: 10.3390/molecules26185651 (PMC8466092; doi:10.3390/molecules26185651)
Supplement: Supplementary file 1 [file molecules-26-05651-s001.zip › molecules-1335033-supplementary.pdf]

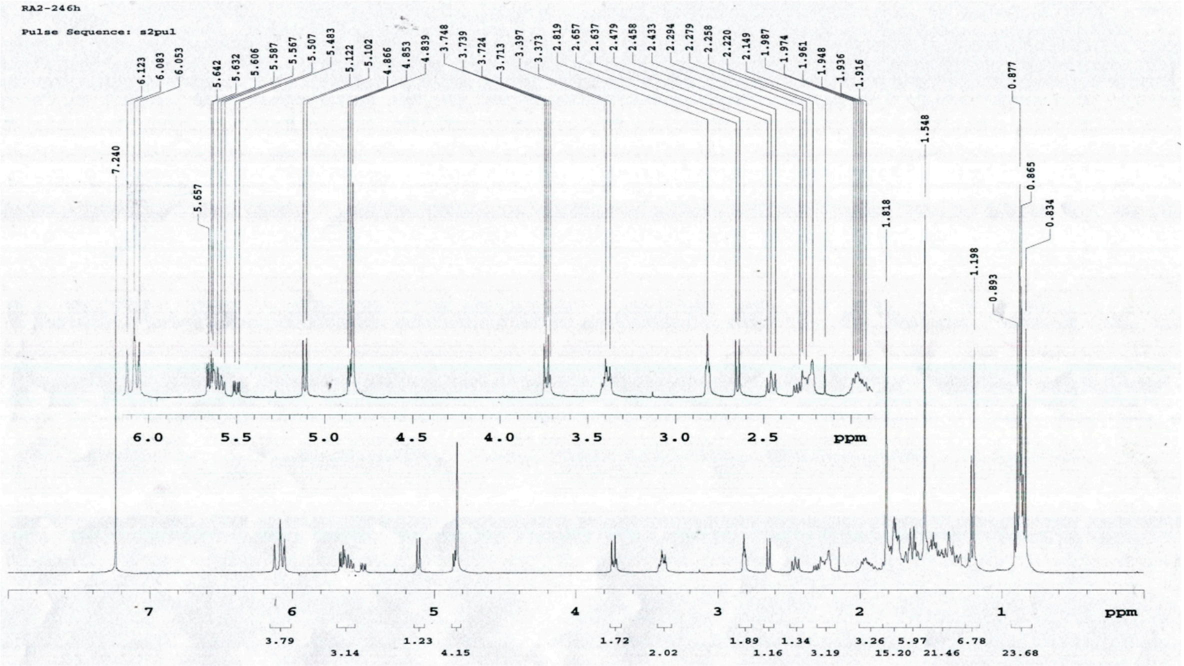
RA2-246h
Pulse Sequence: s2pul
7.240
6.123
6.083
6.053
5.657
5.642
5.632
5.606
5.587
5.567
5.507
5.483
5.122
5.102
4.866
4.853
4.839
3.748
3.739
3.724
3.713
3.397
3.373
2.819
2.657
2.637
2.479
2.458
2.433
2.294
2.279
2.258
2.220
2.149
1.987
1.974
1.961
1.948
1.936
1.916
1.818
1.548
1.198
0.893
0.877
0.865
0.834
6.0
5.5
5.0
4.5
4.0
3.5
3.0
2.5
ppm
7
6
5
4
3
2
1
ppm
3.79
3.14
1.23
4.15
1.72
2.02
1.89
1.16
1.34
3.19
3.26
15.20
5.97
21.46
6.78
23.68

(**a**)

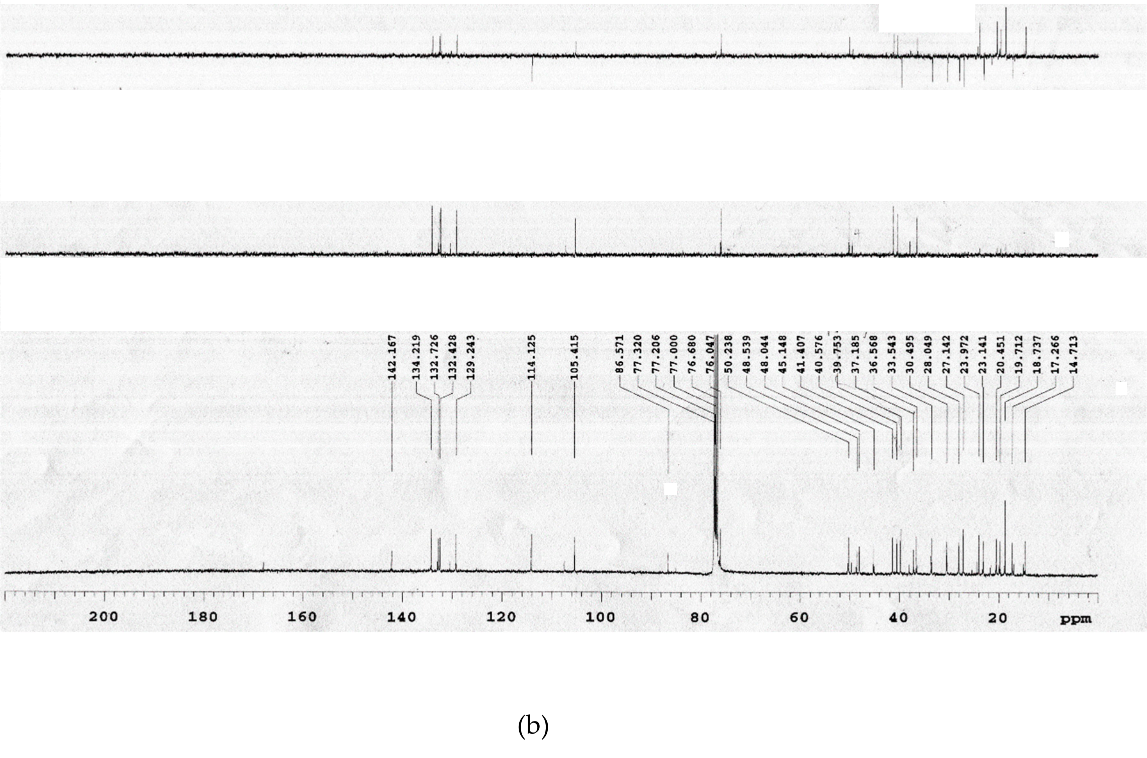
142.167
134.219
132.726
132.428
129.243
114.125
105.415
86.571
77.320
77.206
77.000
76.680
76.047
50.238
48.539
48.044
45.148
41.407
40.576
39.753
37.185
36.568
33.543
30.495
28.049
27.142
23.972
23.141
20.451
19.712
18.737
17.266
14.713
200
180
160
140
120
100
80
60
40
20
ppm

(b)

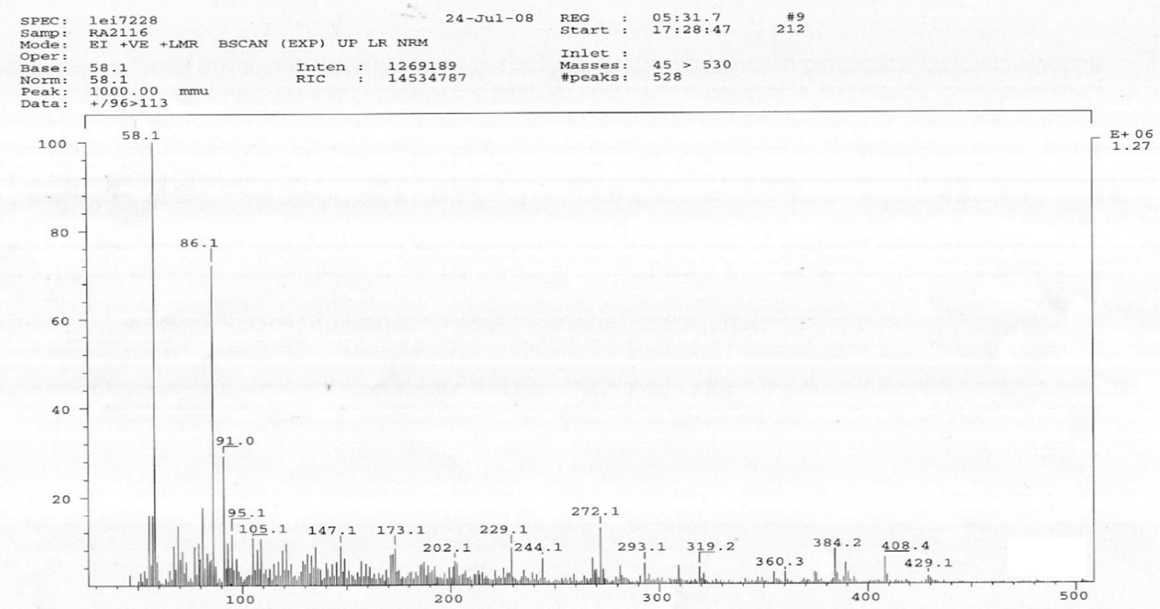

(c)

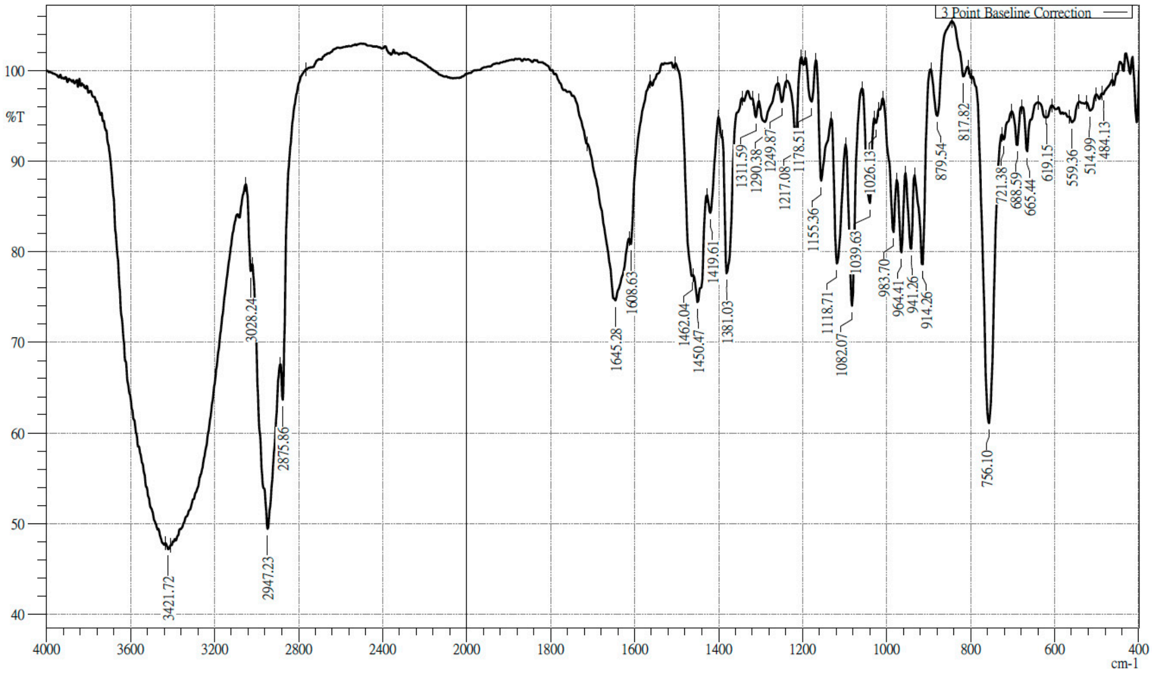

(d)

Figure S1. Spectra of charantadiol A. ^{1}H NMR (400 MHz) of charantadiol A (a), ^{13}C NMR, DEPT-135, DEPT 90 spectra (100 MHz) of charantadiol A (b), EI-MS spectrum of charantadiol A (c), and IR spectrum of charantadiol A (d).
